# Automation of protein crystallization scaleup via Opentrons-2 liquid handling

**DOI:** 10.1016/j.slast.2025.100268

**Published:** 2025-03-16

**Authors:** Jacob B. DeRoo, Alec A. Jones, Caroline K. Slaughter, Tim W. Ahr, Sam M. Stroup, Grace B. Thompson, Christopher D. Snow

**Affiliations:** a School of Biomedical Engineering, Colorado State University, United States of America; b Department of Cellular and Molecular Biology, Colorado State University, United States of America; c Department of Chemical Engineering, Colorado State University, United States of America; d Department of Chemistry, Colorado State University, United States of America; e Department of Electrical Engineering, Colorado State University, United States of America

**Keywords:** Liquid handling, Protein crystallization, Crystallography, Sitting drop crystallization

## Abstract

In this study we present an approach for optimizing protein crystallization trials at the multi-microliter scale utilizing the Opentrons-2 liquid handling robot. Our research demonstrates the robot’s capability to automate 24-well sitting drop protein crystallization trials. Using Python scripts for precise control, the study explores the robot’s application in mixing and setting up crystallization plates with a model protein (hen egg white lysozyme) and a periplasmic protein from *Campylobacter jejuni,* a crystal utilized in the Snow lab as a biomaterial for nanotechnology that requires large, consistent batches. In a head-to-head comparison with manual 24-well plate setup, crystal growth statistics indicate our approach can reduce manual labor and increase reliability in protein crystallization, and may also reduce variability, offering an economical and versatile tool for laboratories. This study shows facile adaption of the Opentrons interface and hardware for growth of two different crystal types. All developed liquid handling routines and relevant data files, in addition to demonstration videos are available at https://github.com/jbderoo/Opentrons2-Protein-Crystallization

## 1. Introduction

Protein crystallization is a key bottleneck for structural characterization via X-ray diffraction. The objective of vapor diffusion crystallization is to induce protein molecules to self-assemble into a precise nanostructure with coherent long-range order [1–3]. These assemblies are crystals, traditionally used for structural biology. Crystal structures often provide key information about protein-protein interactions or critical details to understand protein functions. Observing these features is often essential for rational drug design and validation of computational protein structural prediction [4–7]. Additionally, crystals can be highly porous materials that have recently shown promise as scaffolds for guest molecule structural determination or as biomaterials [8–10]. Both structural biology as well as high-precision biomaterials research benefit from access to repeated growth of large high-quality crystals (“scaleup”).

Protein crystallization is a relatively time-consuming experiment with no guarantee of success. Frequently, large experimental search grids are needed to find crystallization conditions when attempting to crystallize a completely novel protein, and if crystallization conditions are found, optimization of those conditions for repeatable, consistent growth has its own perils. For use of crystals as biomaterials, such as catalytic scaffolds, environmental reservoirs, or drug delivery vehicles, consistent, large batches become more important than for structural biology which may only require a few crystals of sufficient size for X-ray or electron diffraction [11,12]. A nonexhaustive list of parameters frequently varied during crystallization trials include: the identity and concentration of precipitant, pH, buffer type, buffer concentration, protein concentration, and the identity and concentration of any secondary salts [13]. Identifying ideal conditions to induce proteins to crystallize or to influence scale of crystal growth is an iterative, trial and error process. Once crystallization conditions have been identified, scaleup to larger drop sizes that facilitate an increase in crystal size must be optimized as well. We set out to determine if a general-purpose liquid handling system could scaleup crystallization trials (i.e., at the 24-well scale for large growth), including both the reservoir mixing and sitting drop deposition setup steps.

A popular crystal growth strategy is sitting drop vapor diffusion, wherein a volume of concentrated protein is mixed with a volume containing a precipitant [2]. The combined volume is then sealed inside a well containing a separate volume of the undiluted buffer. Gradually, water vapor equilibrates between the sitting drop and the undiluted buffer, ultimately triggering crystal nucleation and growth. Several liquid handling machines exist to help accelerate this brute force process such as the Crystal Gryphon from Art Robbins Instruments (Sunnyvale, CA), the mosquito from SPT Labtech (Melbourn, UK), and the Oryx series from Douglas (Berkshire, UK). However, these machines are not suitable for every protein crystallographer; as standalone instruments they carry maintenance costs and may not be sufficiently economical or versatile for all laboratories.

The Gryphon protein crystallization machine is recognized for its high-throughput capabilities, allowing for the screening of 96 conditions simultaneously [14–21]. It supports various crystallization plate sizes and offers a range of screening blocks, enhancing accessibility for high-throughput screening. However, there are notable drawbacks. Learning a Scratch-like language specific to the Gryphon is required to appropriately build protocols and loop with the Gryphon, and it is necessary to perform stringent cleaning. The preparation or purchasing of deep-well blocks for the protein crystallization conditions must be prepared by hand prior, or an additional and costly attachment to the Gryphon must be purchased to assist in the automation of this step.

Additionally, liquid handling robots can struggle with highly viscous protein solutions, as viscous protein drops tend to accumulate on the pipets, evaporate, and leave solids behind on the tips, thus hindering successful transfer into the wells [15]. Although there are settings to mitigate this, the problem occasionally persists. Ultimately machines that are dedicated to large high-throughput crystallization screens cannot easily be repurposed for other tasks. While the Gryphon streamlines the process of setting up crystallization plates, a major portion of the time savings comes from using pre-made reservoir solution screens. Alternative liquid handling instruments (e.g., Rigaku Alchemist, (Tokyo, Japan)) are needed to automate the creation of such 96-well plates. Given a starting crystallization condition from a high-throughput screen, our focus is the subsequent optimization of crystal growth through tuning the buffer conditions, volumes, and then reliably growing large numbers of the optimized crystals. We refer to this latter phase as “scale up”, and we show here that a general-purpose liquid handling system can favorably compete with manual crystallization trials at this stage (Supplemental Figure 5).

**Fig. 1.**
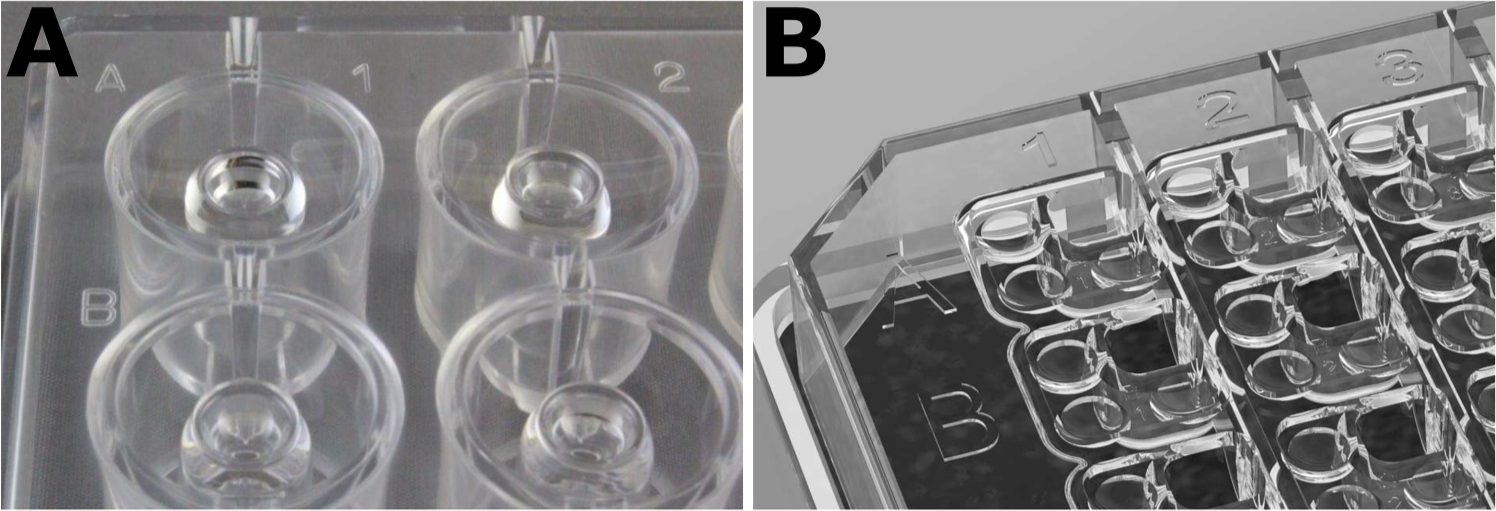
Well geometries for two common protein crystallization plates. **A)** A Hampton Research CrysChem 24-well plate for scaled up sitting drop crystallization. The outer ring “moat” is where the reservoir is prepared. The center raised pedestal contains a large sitting drop well where the reservoir is mixed with the purified protein solution. **B)** A SWISSCI 96-well sitting drop crystallization plate, used for broad crystallization condition screening. Its geometry for vapor diffusion includes 3 small wells with one larger central reservoir. In general, robots designed for preparing initial screening experiments are not compatible with scaleup experiments.

The Opentrons company (Long Island, NY) has comparatively affordable liquid handling robots that are modular in nature [9,22]. Appealingly, the Opentrons robots can be programmed with and execute Python code, providing fine control over all liquid handling steps with many different applications: protein crystallization, serial dilutions, PCR, library prep, ELISAs, extractions, and purifications of protein and DNA. This fine control flexibility can be useful for crystallography, accommodating the diverse schemes used for protein crystallization (e.g., sitting drop, hanging drop, vapor diffusion, and others). Here, we focus on sitting drop experiments with Hampton Research’s CrysChem 24-well plate (AlisoViejo, CA). The geometry of a sitting drop protein crystallization plate is non-trivial, containing both a reservoir moat and a separate pedestal capable of holding a sitting drop of liquid (Fig. 1). An engineering diagram for this plate is also available (Supplemental Figure 1). These plates are larger than standard ANSI/SLAS format and are optimized for the growth of protein crystals of sufficient size for X-ray diffraction structural determination, as well as for manufacturing many large crystals for biomaterial and scaffolding uses. The CrysChem plates have the inherent advantage of being suitable for in situ mixing of a reservoir solution and the crystal growth sitting drop in a single piece of labware, as well as easier visualization and manipulation of synthesized crystals. For comparison, other crystallization screening labware such as the SWISSCI 96-well plate (High Wycombe, UK) is standard ANSI/SLAS format, but is more suited for high throughput discovery of crystallization conditions (as opposed to crystal scaleup) due to its smaller reservoir size. Fortunately, the Opentrons pipets can be moved precisely across the machine deck, allowing pipetting to and from the crystal growth pedestal and the reservoir “moat” for each sitting drop position. Notably, a team at Opentrons demonstrated the OT-2′s ability to create reservoir mixtures via a deep 96-well plate and then fill and plate an MRC Maxi 48-well from SWISSCI. This demonstration is freely available from Opentrons at https://vimeo.com/654672188.

## 2. Materials and methods

### Opentrons-2 liquid handling robot:

Throughout these experiments we used the same Opentrons-2 liquid handling robot (OT-2). The machine was configured with a p10 1st generation pipet arm attached to the left arm, and a p300 2nd generation pipet arm attached to the right arm. 10 μL tips (GEB PT0010–9B-NS) (Shanghai, China) and 200 μL tips (Opentrons 999–00,081) were used for each arm, respectively.

### Custom labware for the OT-2:

**Fig. 2.**
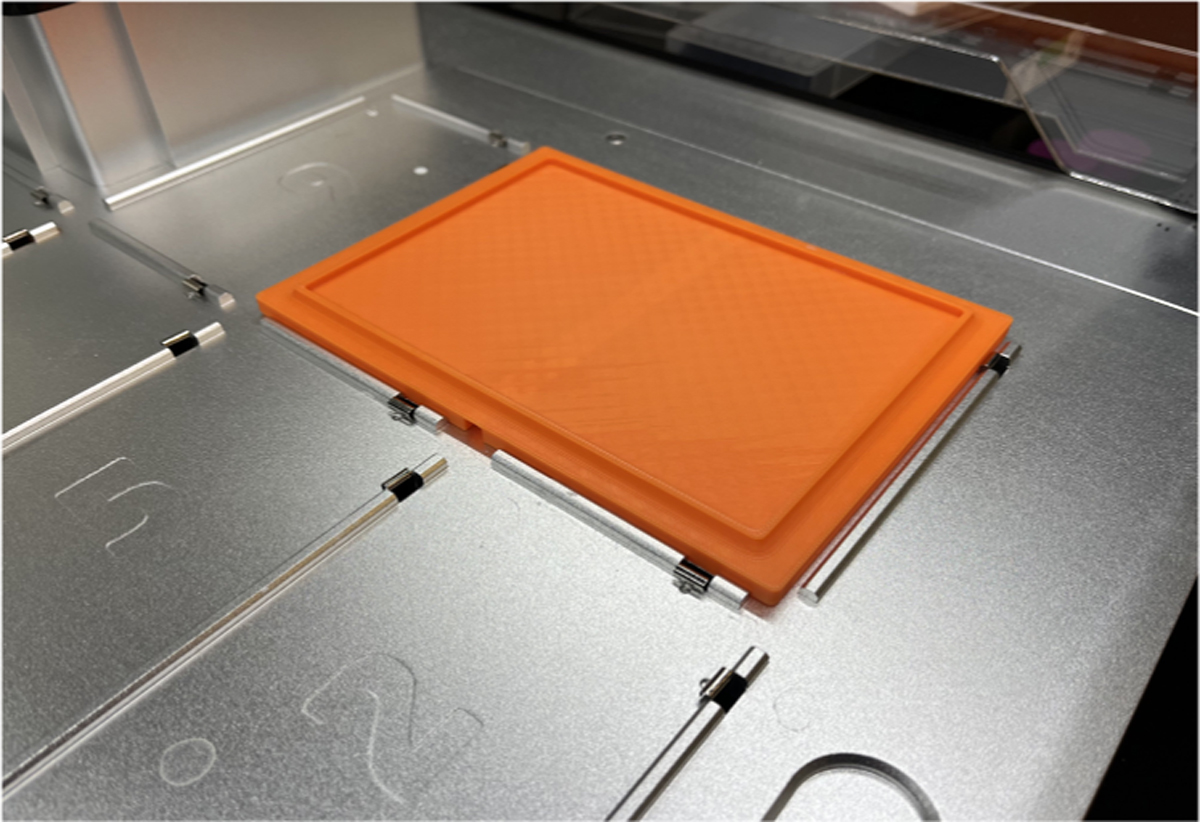
The adapter that holds the 24-well CrysChem plate, 3D printed from the Cryschem_Plate_Adapter_V1.stl file clipped into the Opentrons deck. Notably, there is a notch on the underside such that it mounts the two slots in the deck snuggly. An engineering schematic of this adapter is available at Supplemental Figure 2.

Typical ANSI/SLAS-format laboratory 96-well plates measure approximately 84.5 × 127.8 mm in x and y dimensions. The deck of the OT-2 is preallocated with slots that can lock plates of this size into place so that they cannot move during the experiment. For our tests, we used the HR3–159 Hampton Research CrysChem 24-well plates. These plates are larger than a standard ANSI/SLAS-format plate (150×106 mm). To overcome this barrier, an adapter was designed by the Opentrons engineering team to clip into two of the slots on the OT-2′s deck. The adapter narrows at the top to seat the CrysChem plates and keep them stationary for the duration of the experiment. The adapter was 3D printed on a Bambu Lab X1 Carbon printer (Shanghai, China) using a standard 3D printing material, (poly) lactic acid (from Bambu Lab). The entire print job took 84 min to complete. Fig. 2 shows the final product of this CrysChem sitting drop plate OT-2 adapter. This .stl file can be found at the GitHub for this project (https://github.com/jbderoo/Opentrons2-Protein-Crystallization). This adapter must be 3D printed (or otherwise produced) for use with the CrysChem crystallization plates.

In addition to a physical adapter, the OT-2 needs a data file that tells the robot where the pipet will go in 3D space when location labels are used. For example, when told to go to “A1”, the arm will go to 30.5 mm in the x direction, 12.5 mm in the y direction, and 6.1 mm in the z direction. This .json file is also available on the GitHub, and after downloading must be uploaded as a custom labware definition to the OT-2. Members of the Opentrons support team were incredibly helpful in the troubleshooting and development of both files. The custom labware definition was further refined by the authors.

### Python scripts:

Opentrons liquid handling robots are programmable via Python, allowing both fine control of the robot and accessible customization to new projects via easy adaptation of starter scripts. All scripts written for this project were written in Python 3.9 with the Opentrons Python module. For more information about the package and installation, see https://docs.opentrons.com/v2/ and https://anaconda.org/conda-forge/opentrons respectively.

Three example scripts (discussed individually in subsequent sections) are each divided into a few major sections, and are available on the GitHub. (1) The first section of each script describes the geometry of the source tubes to prevent the OT-2 from completely submerging the pipet into the source liquid. (2) The second section of each script describes the volume of every source liquid that must be transferred into every well on the CrysChem plate. (3+) The remaining sections describe how to transfer liquid to the intended destinations in a sitting drop plate.

### Script 1, mixing demonstration with food dye:

**Fig. 3.**
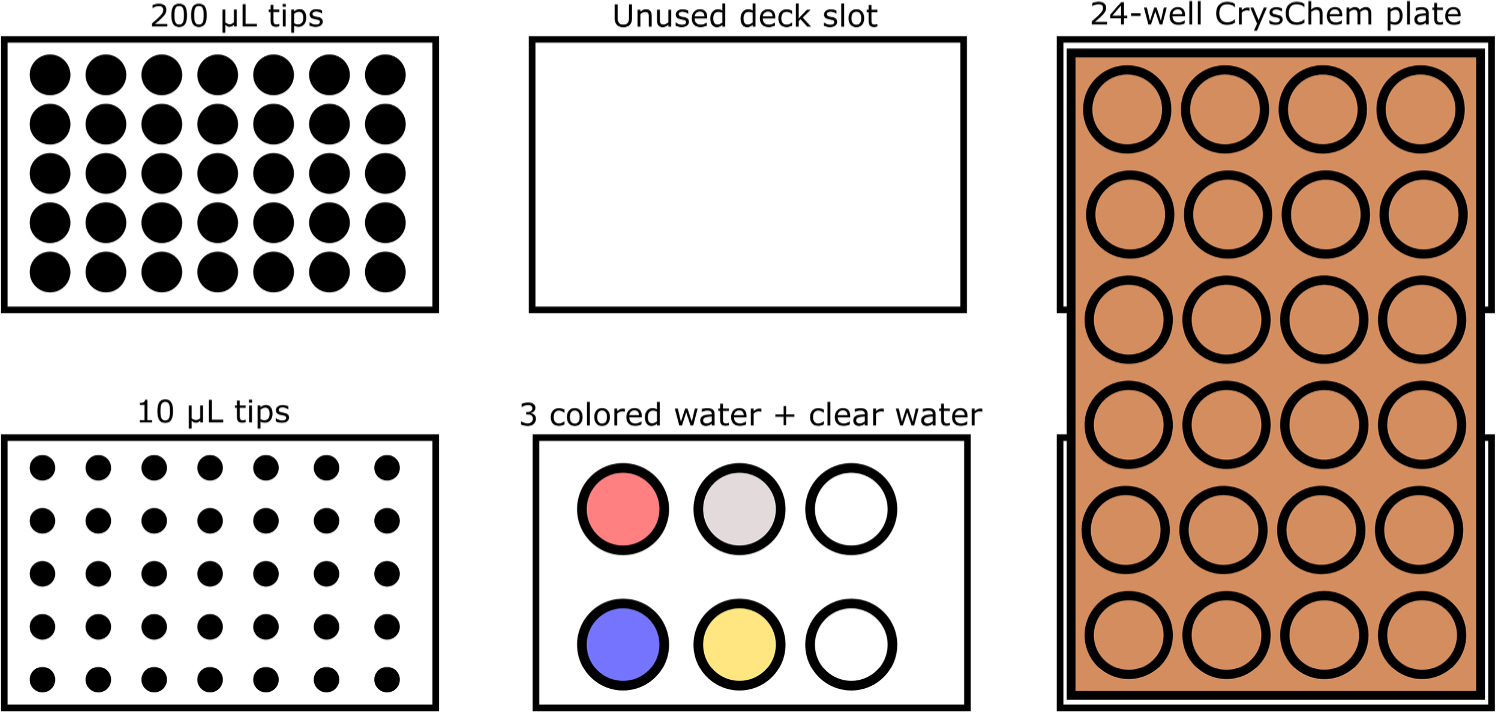
A cartoon schematic of the setup inside the OT2 to prepare the color gradient plate. For a full diagram of the Python script’s movements, see Supplemental Figure 3.

To mimic a conventional sitting drop 24-well experiment, we programmed the OT-2 to mix a variety of candidate buffers (blue and red food dye in water) in the reservoir sections of the plate. An increasing amount of blue dye was added every column (0–150 μL from a pure blue stock solution, stepping by 30 μL) and an increasing amount of red dye was added every row (0–150 μL from a pure red stock solution, stepping by 50 μL). The remainder of each reservoir was filled with water to a total volume of 400 μL. Upon reservoir completion, 5 μL of yellow dye stock solution was aspirated, then 5 μL of the reservoir was aspirated in the same pipet tip, bringing the in-tip volume to 10 μL. This volume was then dispensed into the corresponding pedestal. Doing this in a single step did not significantly contaminate any important liquids, and saved both time and number of tips. Fig. 3 highlights the deck set up required for these steps, and Supplemental Figure 3 includes arrows depicting the flow of source liquid to destination as a pictorial representation of the scripts.

### Script 2, Hen Egg White Lysozyme (HEWL) crystallization:

HEWL crystals were synthesized using several different stock solutions: (1) Hampton Research’s premixed “15-Minute Lysozyme Crystallization Reagent” (HR2–805), (2) Hampton Research’s purified lysozyme protein (HR7–110) at 20 mg/mL in a 20 mM sodium acetate (Sigma Aldrich, St. Louis, MO) buffer at a pH of 4.6, and (3) a 200 mM sodium acetate stock solution of varying pH; (3a) 4.6, (3b) 4.7, and (3c) 4.8. A 24-well plate was set up where each crystallization trial used 3 stock solutions. To prepare the reservoirs, 300–350 μL of solution (1) was added to every column (increasing by 10 μL every column). 50 μL of solution (3a) was added to each well of row A, 50 μL of solution (3b) to each well of row B, and 50 μL of solution (3c) to each well of rows C and D. To bring every reservoir up to 400 μL, DI water was added as necessary (Supplemental Figure 4 A). After all the reservoirs were mixed (discussed momentarily in the Results and Discussion section), the pedestal sitting drops were formed. 2 μL of solution (2) was aspirated into the pipet. This was immediately followed by aspiration of 2 μL of an individual well’s reservoir fluid. The 4 μL sitting drop volume was then dispensed into the elevated crystal formation pedestal. This process is then repeated for each individual well. We then manually sealed the plate with Duck HP260 (Avon, OH) clear packaging tape after all 24 sitting drops had been prepared.

### Script 3, CJ crystallization:

The CJ protein is an engineered variant of CJ0420, a periplasmic protein of unknown in vivo function from *Campylobacter jejuni*. CJ crystals were grown according to previously optimized conditions [23]. The crystal structure is known (PDB entry: 5W17). A CrysChem 24-well plate was set up using three stock solutions: (1) 1 M Bis-Tris (RPI, Mt Prospect, IL) at pH 6.0 or 6.5, (2) 4 M ammonium sulfate (Fisher Chemical, Pittsburg, PA), and (3) CJ protein at 12 mg/mL in 50 mM HEPES (VWR Life Science, Radnor, PA), 500 mM ammonium sulfate, 10 % glycerol (Fisher Biogreagents, Pittsburg, PA) at pH 7.4. Stock solution (3) was aliquoted in bulk into 100 μL batches in 1.5 mL low adhesion microcentrifugation (USA Scientific, Ocala, FL) tubes. 40 μL of solution (1) was added to every well; rows A and C were pH 6.0, and rows B and D were 6.5. Between 335 μL and 360 μL of solution (2) (stepping 5 μL per column) was added to every well (Supplemental Figure 4 B). Water was added to bring the total volume of each reservoir to 400 μL. After all the reservoirs were mixed (discussed in subsequent section), 2 μL of stock (3) was aspirated into the pipet. Immediately after, 2 μL of the reservoir was aspirated. The 4 μL sitting drop volume was then dispensed into the elevated crystal formation pedestal. We then manually sealed the plate with Duck HP260 clear packaging tape after all 24 sitting drops had been prepared.

## 3. Results and discussion

Overall, the OT-2 was able to achieve the desired goals of mock and authentic crystal plate creation after minimal trial and error. Issues such as viscosity were identified and addressed. Evaluation experiments included: Script 1: preparation of a color gradient, Script 2: 24-well HEWL crystallization, Script 3. 24-well CJ crystallization.

### Script 1:

**Fig. 4.**
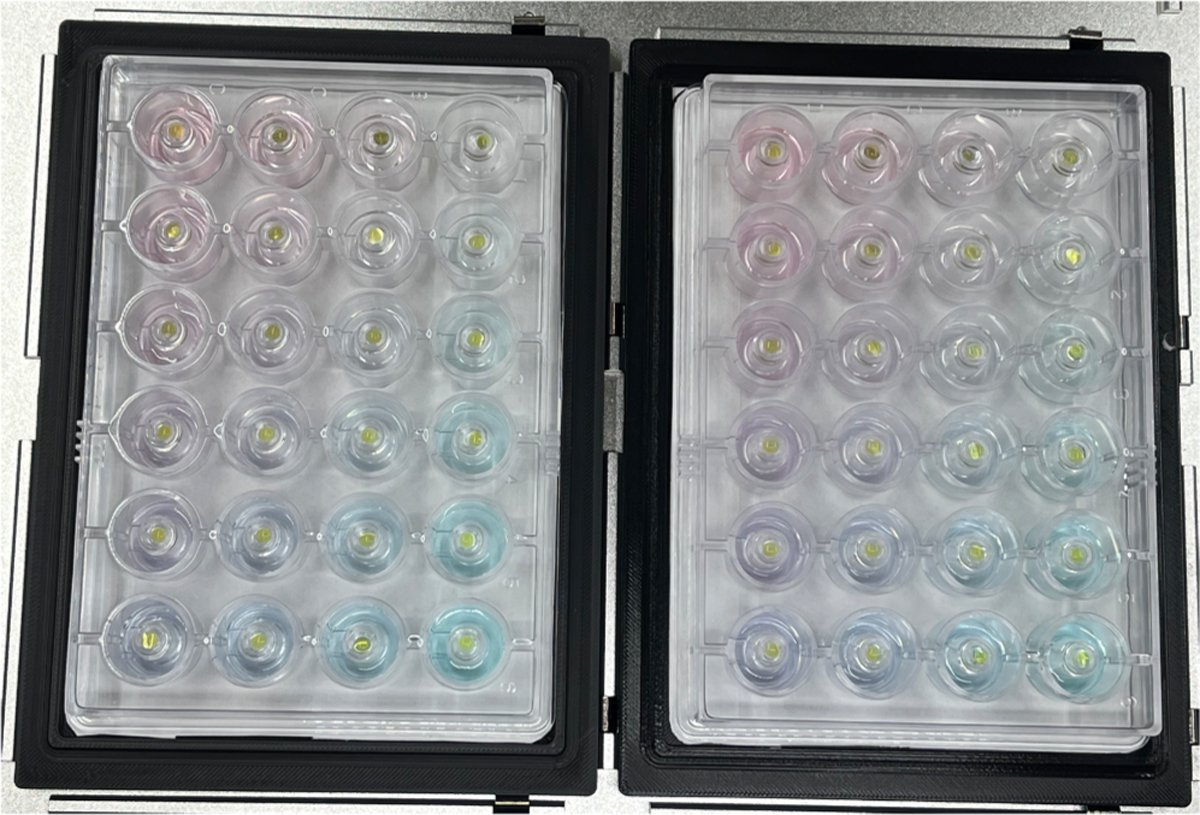
Proof of concept sitting drop plates with food coloring. The OT-2 prepared a sitting drop plate with dyes to visualize correct mixing (left) compared to an experimentalist’s sitting drop plate with dyes (right). A blue gradient increases from left to right, and a red gradient increases from top to bottom. Each reservoir mixture was then mixed with a yellow dye stock to mimic combining each reservoir solution with purified protein in a sitting drop.

As an initial proof of concept, we used food dye (red, blue, yellow) and colorless water to visualize the process of setting up a crystal plate. This strategy is highly recommended per Opentrons when developing liquid handling scripts to trouble shoot any errors that may arise and to ensure reagents are going to the intended locations. A gradient of red food dye was increased as we moved down the plate (more red dye in row D, less red dye in row A). A gradient of blue dye was increased as we moved across the plate (more blue dye in column 6, less blue dye in column 1). The process was repeated by manual pipetting, and no differences were discernable (Fig. 4). The wells were correctly constructed with red, blue, and clear water. Importantly the sitting drops were prepared correctly by combining 5 μL yellow dye (standing in for the biomolecules) with 5 μL from reservoir. From this process, we learned that an additional step was needed for the OT-2 to mimic manual pipetting. Specifically, at the 400 μL volume, the reservoir solutions can have a sufficiently high contact angle and low volume that prevents uniform dispersion around moat volume when all reservoir components were dispensed at the top of the reservoir (i.e. at the 12:00 position on a clock). This effect was stochastic, affecting 19 of the reservoirs out of 24 total. In so far as this effect represents a potential source of variability for vapor-liquid equilibrium, we proceeded to develop a surface flattening/mixing technique. To this end, another step was added that withdrew 200 μL from the 12:00 position of the reservoir, then moved to the 3:00 position and dispensed 22 μL, then moved to the 4:00 position and dispensed 22 μL, and was repeated until the distributed dispensing steps no longer held any volume. In most cases this extra step successfully overcame the viscosity, causing the reservoir liquid to be uniformly distributed around the central sitting drop pedestal. Notably, a second and more time efficient but more hands-on approach is adaptable; we can easily add a command for the robot to pause and wait for a user’s input to proceed. While this requires someone to set an alarm and walk over to the OT2 (thus interrupting their workflow) to gently swirl the plate, it greatly reduces the mixing process time from 9 min to 30 s. The time required for the OT-2 to construct this plate was 27 min. 27 min does not include the 9 min required to have the OT2 to mix the reservoir; instead, we opted to gently swirl the CrysChem plate manually. It took approximately 15 min to prepare the reservoir solutions and 12 min to prepare the sitting drops.

For clarity, we have also included a short YouTube video of our OT-2 preparing the major diagonal of the color plate for visualization, available at our GitHub.

### Script 2:

**Fig. 5.**
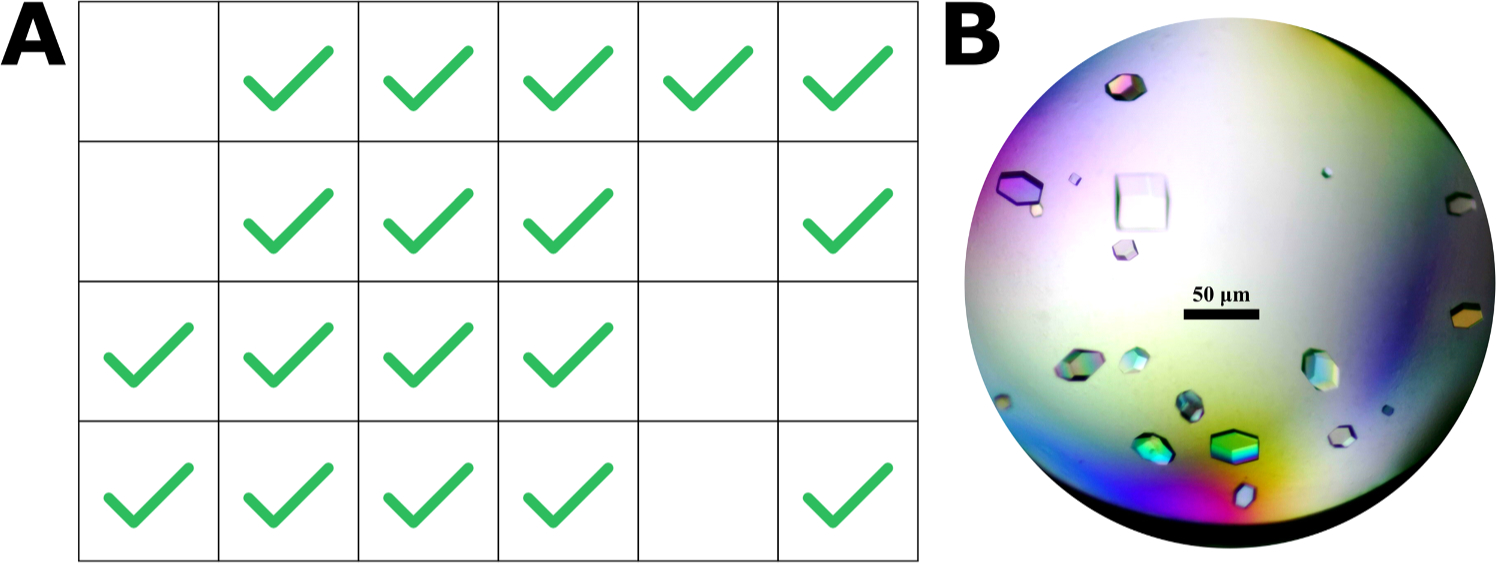
Crystallization results from the OT-2 HEWL 24-well plate sitting drop. **A)** A cartoon schematic of the CrysChem plate, where green check marks highlight successfully grown HEWL crystals. 18/24 wells successfully yielded HEWL crystals in 24 h. **B)** One of the wells (D4) that successfully yielded HEWL crystals.

With a proof of concept established, we moved onto HEWL, a protein that readily crystallizes under suitable conditions [24–26]. After 24 h crystals had formed in 18 out of 24 wells (Fig. 5). Similar pipetting techniques to the food dye were employed in the HEWL script, with two notable differences. First, the aspirating and redispensing of 200 μL at 3:00–9:00 was not needed due to a difference in contact angle of the crystallization media. Second, a “tip shaking” step was added to ensure the purified protein:well reservoir mixture was completely removed from the pipet tip; the pipet tip quickly touches the wall at the 12:00, 3:00, 6:00, and 9:00 positions if the wall of the well was a clock. In our group, a 2 μL total sitting drop volume is most common when growing many large protein crystals (scaleup crystal production). We tested the OT-2 script at this volume but found the OT-2 with the Gen1 p10 pipet could not consistently eject the 2 μL onto the sitting drop pedestal. Frequently, the protein:reservoir mixture was too “sticky” to leave the tip and arrive at the bottom of the pedestal, and instead would curl up the outside of the pipet tip, even with the pipet tip shake step. Doubling the final total sitting drop volume from 2 μL to 4 μL total (as described previously) rectified this issue. The time required for the OT-2 to construct this plate was 31 min. The crystallization media flowed fully and completely in the reservoir and did not require a mixing step or a pause for a manual swirling of the plate. It took approximately 12 min to create the sitting drops.

**Fig. 6.**
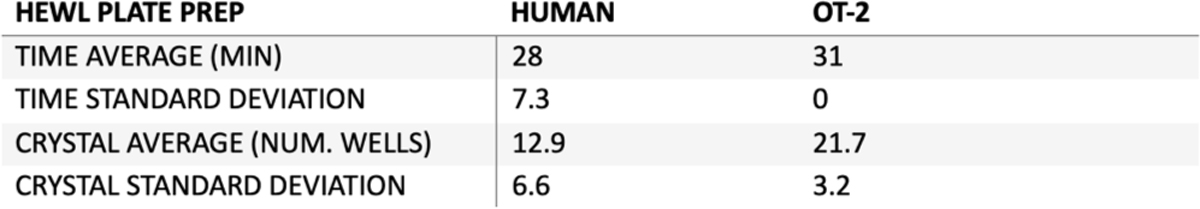
Final results of the human vs OT-2 HEWL plate preparation. For full data, see Supplemental Figure 5.

To better assess the capabilities of the OT-2 with regards to protein plate production, we prepared two additional plates with the OT-2, then nine more plates prepared by three different people with varying wet lab familiarity. Although the OT-2 prepares plates slightly slower than your average person (and statistically-significantly slower than an experienced wet lab scientist), it frees a researcher for approximately 30 min and offers the key advantage of more consistently producing protein crystals (Fig. 6, Supplemental Figure 5). To further test the OT-2′s abilities, we also sought to test accuracy and precision when pipetting small volumes of water. At volumes of 1 and 100 μL, we found no statistically significant difference between pipetting with the OT-2 and a human being (Supplemental Figure 6). Lastly, we note that many protein crystallization buffers include high-viscosity components. Opentrons reports the volume loss expected when pipetting highly viscous solutions (99 %, 90 %, and 10 % glycerol) to be 0.9 % for the 20 μL Gen2 Single-Channel Pipet and 0.2 % for the 300 μL Gen2 Single-Channel Pipet [27]. However, given the importance of volume accuracy we sought to verify high-viscosity pipetting accuracy. Specifically, we quantified error for 0 %, 10 %, 20 %, 40 %, 60 %, 80 %, and 100 % glycerol. As expected, the highest mean average percent error was pipetting 1 μL of 100 % glycerol, for which we calculated 13.5 %. At more moderate viscosity (10 % to 40 % glycerol), errors dropped to 0.9 % - 6.2 % for the 10 μL Gen1 Single-Channel Pipet, and 1.6 % - 3.2 % for the 300 μL Single-Channel Pipet (Supplemental Figure 7).

### Script 3:

**Fig. 7.**
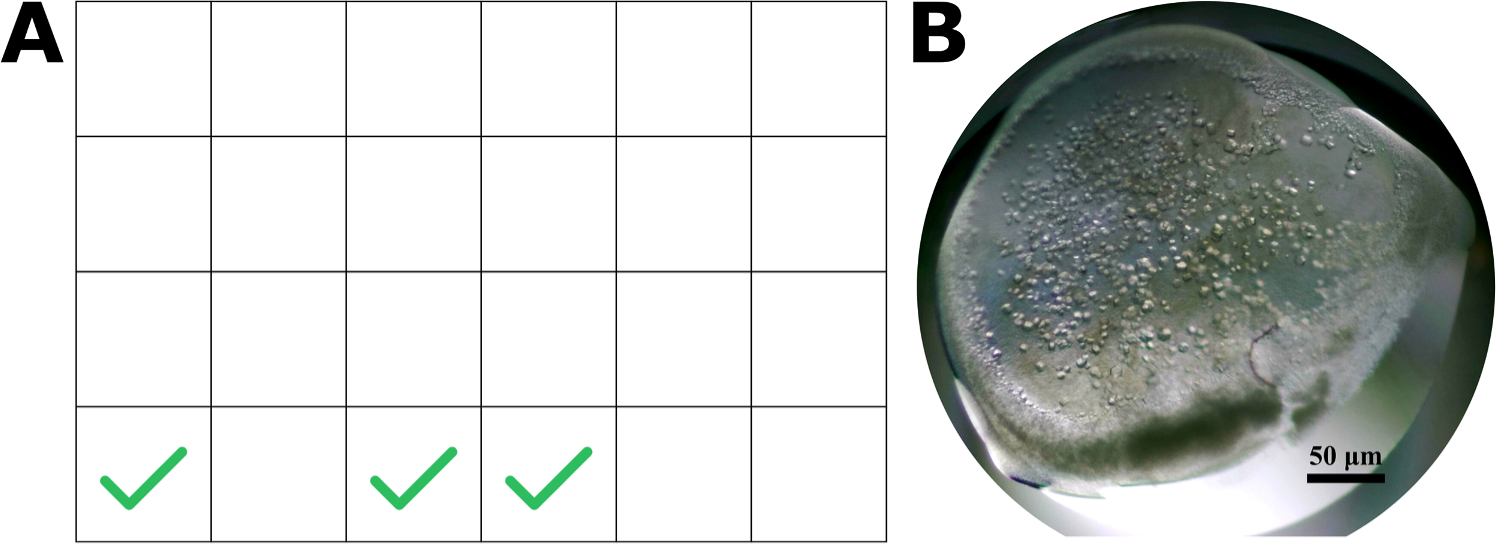
Crystallization results from the OT-2 making a CJ 24-well plate sitting drop. **A)** A cartoon schematic of the CrysChem plate, where green check marks highlight successfully grown CJ crystals. 3/24 wells yielded CJ crystals in 24 h. **B)** One of the wells (D3) that yielded CJ microcrystals.

With a solid grasp of the OT-2′s capabilities and a script foundation, we next sought to demonstrate automated crystallization of a protein we have extensively crystallized in the past [23]. CJ crystals have unusually large solvent channels, enabling intra-crystal macromolecule transport, leading to diverse applications such as textile conjugation [28], enzyme immobilization [8], and mosquito tracking [29]. A 24-well CrysChem plate was prepared, with two buffers at pH 6.0 and 6.5 and with an ammonium sulfate salt concentration ranging from 3.35 M – 3.60 M. CJ crystals typically grow in 24 – 72 h, but can take as long as several weeks to form. After 24 h, CJ microcrystals had formed with the expected, signature hexagonal habit in three wells (Fig. 7). The reservoir surface flattening/mixing technique was employed. Tip shaking was not needed in this case. A total sitting drop volume of 4 μL was used, similar to the HEWL plate. The time required for the OT-2 to construct this plate was 40 min. This time included the additional 9 min to have the OT-2 do the mixing process for us. It took approximately 12 min to create the sitting drops.

## 4. Conclusion

In this study, we presented an economical and easy to use liquid handling protocol that enables fine control over pipetting via Python scripts to construct mock and authentic 24-well sitting drop protein crystal plates with colored water, HEWL, and CJ protein. While 35 – 40 min is not a fast setup time compared to a trained scientist or technician, it does eliminate the need for a trained technician to create these plates. A lab scientist is only needed for setup, plate sealing with tape, and tear down. Perhaps more importantly, we suspect robotic plate setup will greatly reduce plate variability from person to person.

Crystallization trials that require volatile components may not be suitable for lengthy plate setup times, though it is relatively easy to insert pauses into the script for manual sealing of individual wells. Alternatively, the script could easily be modified to pause after setting up a row of sitting drops, allowing the attending scientist to seal the completed wells. Additionally, a humidity controlling device (e.g., the MiTeGen Watershed (Ithaca, NY)) could be used to humidify the interior of the OT-2, limiting variability due to sitting drop evaporation. A single OT-2 costs $13,500 at the time of this writing, significantly cheaper than a Gryphon machine priced at $55,000-$75,000. With the adapter, custom module, and starter scripts presented in this paper, the protein structure community at large could affordably test scaleup sitting drop plates at the 4 μL sitting drop scale. Notably, the new Opentrons Flex instrument reportedly has precision down to 1 μL ± 0.13 μL water, which may allow future users to reliably adapt the approach described here to crystallization trials smaller than the 4 μL sitting drops described here. The use of Python scripts for robot programming are a large advantage moving forward since it maximizes accessibility to the largest number of researchers, and facilitates help from large language models for non-expert programmers.

That said, it is important to note that there are many liquid handling automation systems, and the advantages that we have discussed for applying a general pipetting system for crystallization trials are not exclusive to Opentrons. General purpose liquid handling robots could also prove useful for other screen preparation steps in service of biomolecular crystallography, as well as non-crystallographic liquid handling needs. A general-purpose liquid handling robot’s ability to adapt with the needs of a lab significantly reduces the cost burden for automation of multiple laboratory processes. This study showcases the potential for automated systems to enhance protein crystallization workflows, making liquid handling for protein crystallography more accessible.

Research reported in this publication was supported by NIAID of the National Institutes of Health under award number 1R01AI168459-O1A1.

## 5. Declaration of AI-assisted technologies

During the preparation of this work the author(s) used ChatGPT-4 in order to combine and condense multiple paragraphs written about the Crystal Gryphon by multiple people as well as prepare an initial abstract. After using this tool/service, the author(s) reviewed and edited the content as needed and take(s) full responsibility for the content of the publication.

## Supplementary Material

SI

Supplementary materials

Supplementary material associated with this article can be found, in the online version, at doi:10.1016/j.slast.2025.100268.
