# Supplementary material for: Automation of protein crystallization scaleup via Opentrons-2 liquid handling
Source: SLAS Technol. Author manuscript; Available in PMC 2025 Jul 6. (PMC12229254; doi:10.1016/j.slast.2025.100268)
Supplement: SI [file NIHMS2089719-supplement-SI.pdf]

Supplemental Figures

Supplemental Figure 1: Engineering schematic of the CrysChem plate from Hampton Research

Supplemental Figure 2: Engineering schematic of the CrysChem adapter from Opentrons

Supplemental Figure 3: Cartoon visualization of the color plate prep walk through

Supplemental Figure 4: Contents of both the HEWL and CJ 24-well plate CrysChem plates

Supplemental Figure 5: Statistics of OT-2 vs Human HEWL preparation via 24-well CrysChem plate

Supplemental Figure 6: Statistics of OT-2 vs Human small volume water pipetting

Supplemental Figure 7: Accuracy and Precision of OT-2 pipetting liquids with varying viscosity

Supplemental Figure 1

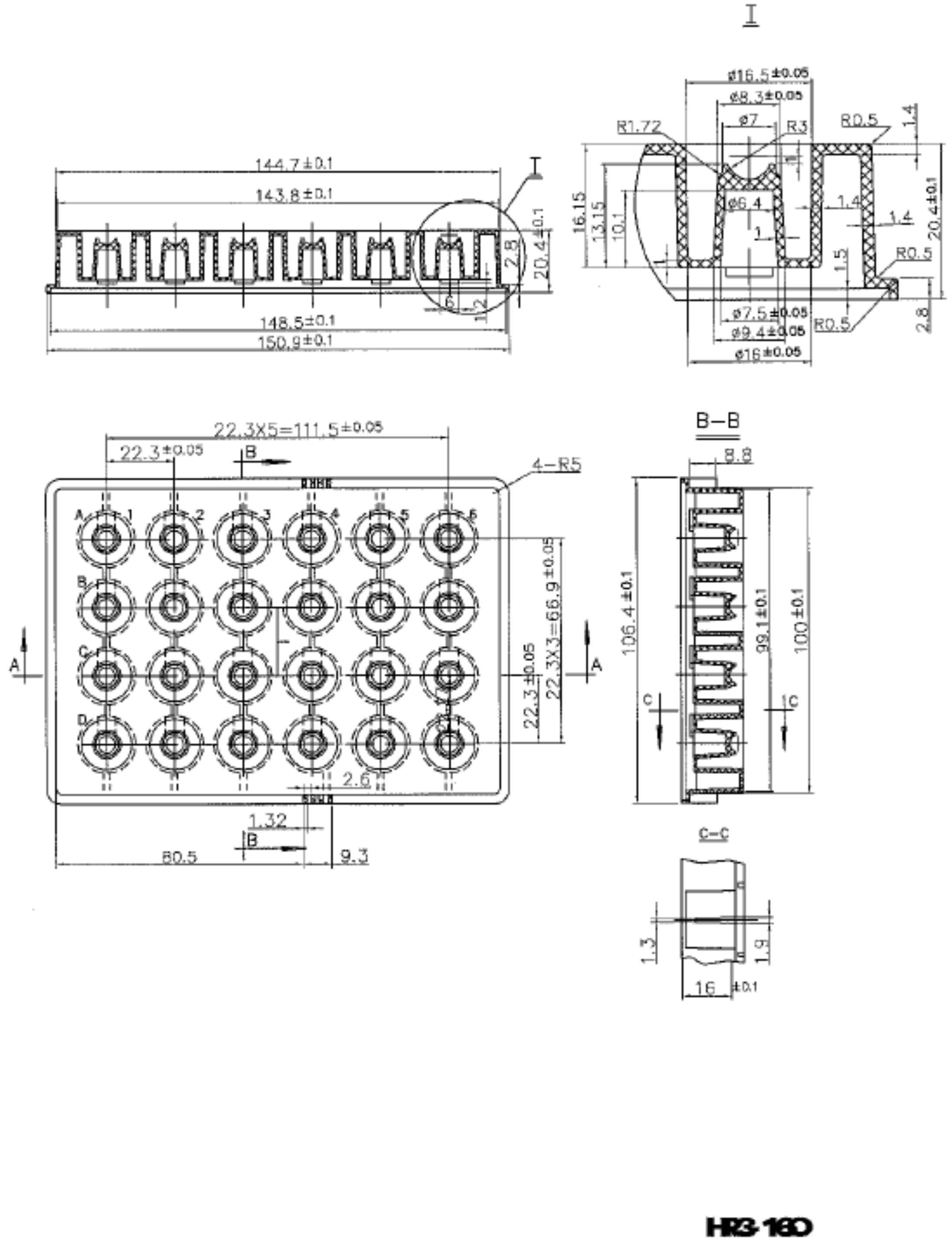

Engineering schematic of the 24-well CrysChem Plate from Hampton Research.

Supplemental Figure 2

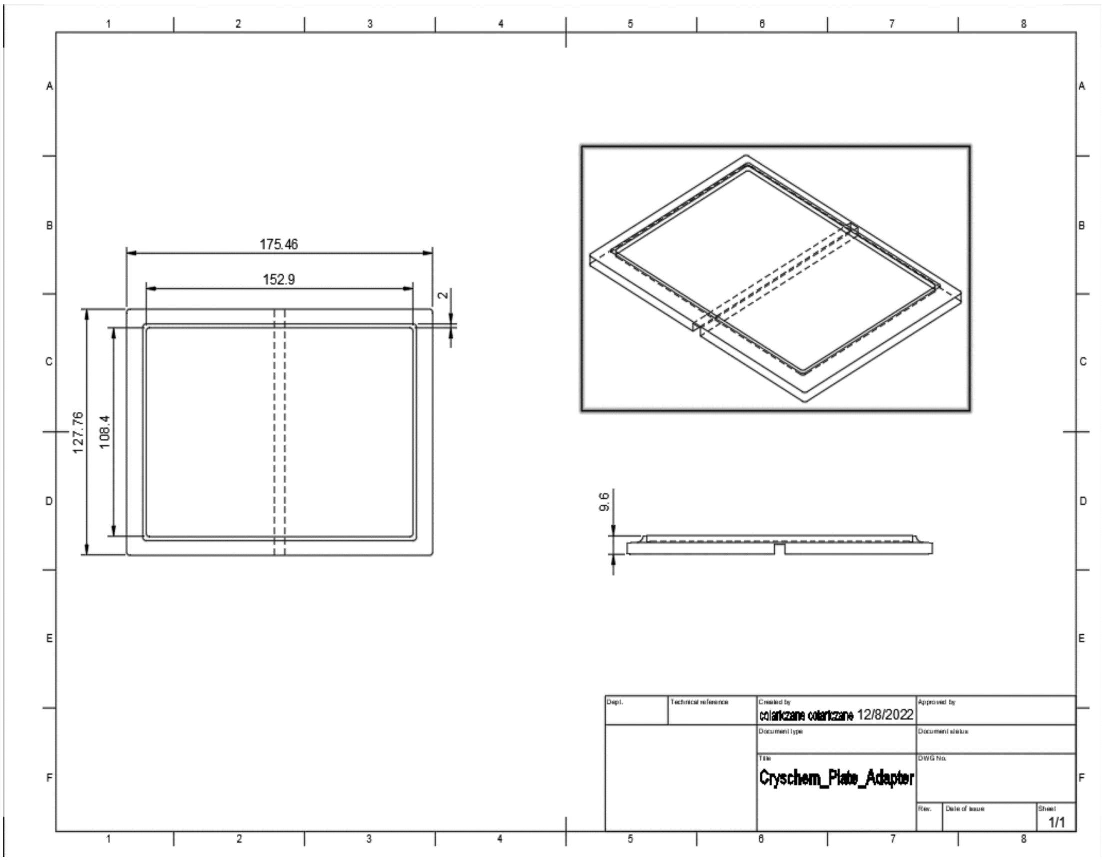

The engineering schematic for the CrysChem 24-well plate adapter that fits into two of the OT2’s deck slots.

1 Supplemental Figure 3

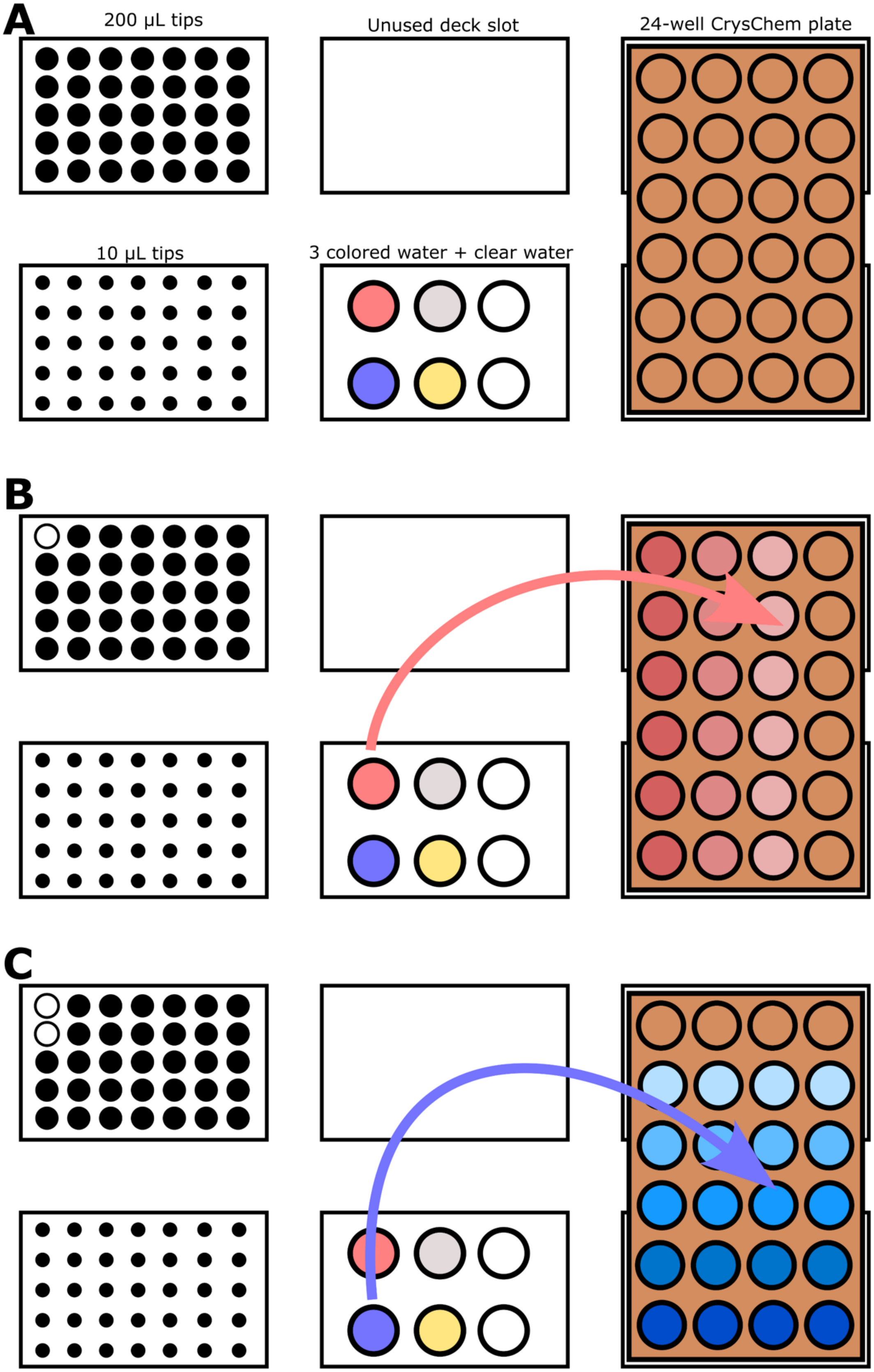

2

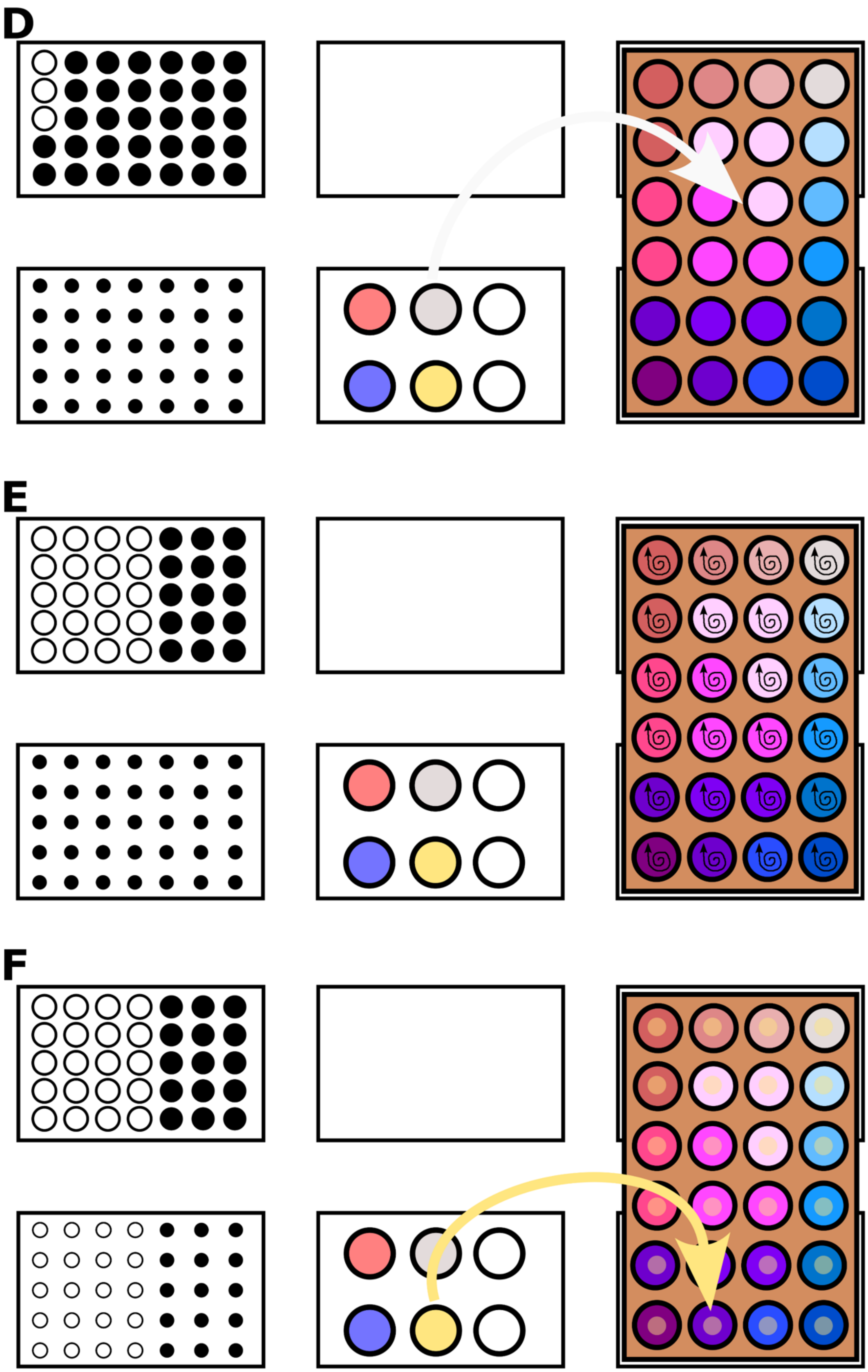
D
E
F

A walk through of the software, specifically *Script 1* (in which a blue-red color gradient is made). **A)** The starting configuration of the OT-2 deck. Deck slots are numbered left to right, bottom up. In deck slot 1 is 96 - 10 µL tips; in deck slot 2 are 4 colors of water (red, clear, blue, and yellow); in deck slot 3 and 6 is the Crys Chem adapter that fits the crystallization plate; in deck slot 4 is 96 - 200 µL tips; deck slot 5 is empty. **B)** A 200 µL tip is grabbed, and used to transfer an increasing amount of red water into the protein crystallization plate. **C)** A new 200 µL tip is grabbed, and is used to transfer and increasing amount of blue water (transposed to the red water) to the protein crystallization plate. **D)** A new 200 µL tip is grabbed, and is used to transfer a variable amount of water to every reservoir, raising the total volume to 400 µL inside the reservoir. **E)** A new tip is grabbed, and is used to mix the reservoir. This tip is discarded, and a new one is acquired, using 24 tips in total. Optionally, this step can be skipped, and the robot can be paused and wait for human input; this would allow for someone in the lab to step in, manually gently swirl the plate, then let the robot resume at step F. **F)** A new tip is grabbed, and yellow water is then aspirated. Then an equal amount of mixed reservoir solution is aspirated. This combination is then dispensed onto the pedestal of the CrysChem plate.

Supplemental Figure 4

A

HEWL plate prep		Column 1	Column 2	Column 3	Column 4	Column 5	Column 6
Row A	pH 4.6	50 µL buffer<br>300 µL HR2-805<br>50 µL water	50 µL buffer<br>310 µL HR2-805<br>40 µL water	50 µL buffer<br>320 µL HR2-805<br>30 µL water	50 µL buffer<br>330 µL HR2-805<br>20 µL water	50 µL buffer<br>340 µL HR2-805<br>10 µL water	50 µL buffer<br>350 µL HR2-805<br>0 µL water
Row B	pH 4.7	50 µL buffer<br>300 µL HR2-805<br>50 µL water	50 µL buffer<br>310 µL HR2-805<br>40 µL water	50 µL buffer<br>320 µL HR2-805<br>30 µL water	50 µL buffer<br>330 µL HR2-805<br>20 µL water	50 µL buffer<br>340 µL HR2-805<br>10 µL water	50 µL buffer<br>350 µL HR2-805<br>0 µL water
Row C	pH 4.8	50 µL buffer<br>300 µL HR2-805<br>50 µL water	50 µL buffer<br>310 µL HR2-805<br>40 µL water	50 µL buffer<br>320 µL HR2-805<br>30 µL water	50 µL buffer<br>330 µL HR2-805<br>20 µL water	50 µL buffer<br>340 µL HR2-805<br>10 µL water	50 µL buffer<br>350 µL HR2-805<br>0 µL water
Row D	pH 4.8	50 µL buffer<br>300 µL HR2-805<br>50 µL water	50 µL buffer<br>310 µL HR2-805<br>40 µL water	50 µL buffer<br>320 µL HR2-805<br>30 µL water	50 µL buffer<br>330 µL HR2-805<br>20 µL water	50 µL buffer<br>340 µL HR2-805<br>10 µL water	50 µL buffer<br>350 µL HR2-805<br>0 µL water

B

CJ plate prep		Column 1	Column 2	Column 3	Column 4	Column 5	Column 6
Row A	pH 6.0	40 µL buffer<br>335 µL $(NH_4)_2SO_4$<br>25 µL water	40 µL buffer<br>340 µL $(NH_4)_2SO_4$<br>20 µL water	40 µL buffer<br>345 µL $(NH_4)_2SO_4$<br>15 µL water	40 µL buffer<br>350 µL $(NH_4)_2SO_4$<br>10 µL water	40 µL buffer<br>355 µL $(NH_4)_2SO_4$<br>5 µL water	40 µL buffer<br>360 µL $(NH_4)_2SO_4$<br>0 µL water
Row B	pH 6.5	40 µL buffer<br>335 µL $(NH_4)_2SO_4$<br>25 µL water	40 µL buffer<br>340 µL $(NH_4)_2SO_4$<br>20 µL water	40 µL buffer<br>345 µL $(NH_4)_2SO_4$<br>15 µL water	40 µL buffer<br>350 µL $(NH_4)_2SO_4$<br>10 µL water	40 µL buffer<br>355 µL $(NH_4)_2SO_4$<br>5 µL water	40 µL buffer<br>360 µL $(NH_4)_2SO_4$<br>0 µL water
Row C	pH 6.0	40 µL buffer<br>335 µL $(NH_4)_2SO_4$<br>25 µL water	40 µL buffer<br>340 µL $(NH_4)_2SO_4$<br>20 µL water	40 µL buffer<br>345 µL $(NH_4)_2SO_4$<br>15 µL water	40 µL buffer<br>350 µL $(NH_4)_2SO_4$<br>10 µL water	40 µL buffer<br>355 µL $(NH_4)_2SO_4$<br>5 µL water	40 µL buffer<br>360 µL $(NH_4)_2SO_4$<br>0 µL water
Row D	pH 6.5	40 µL buffer<br>335 µL $(NH_4)_2SO_4$<br>25 µL water	40 µL buffer<br>340 µL $(NH_4)_2SO_4$<br>20 µL water	40 µL buffer<br>345 µL $(NH_4)_2SO_4$<br>15 µL water	40 µL buffer<br>350 µL $(NH_4)_2SO_4$<br>10 µL water	40 µL buffer<br>355 µL $(NH_4)_2SO_4$<br>5 µL water	40 µL buffer<br>360 µL $(NH_4)_2SO_4$<br>0 µL water

A matrix representation of the CrysChem 24-well plate sitting drop used to synthesize **A)** HEWL and **B)** CJ crystals.

Supplemental Figure 5

	Person 1			Person 2			Person 3			Opentrons 2		
	Plate 1	Plate 2	Plate 3	Plate 1	Plate 2	Plate 3	Plate 1	Plate 2	Plate 3	Plate 1	Plate 2	Plate 3
Minutes	30	22	24	47	38	24	25	22	20	31	31	31
Tips used	29	29	29	43	43	43	32	35	35	37	37	37
Num mistakes	0	1	0	0	0	0	0	0	0	0	0	0
Crystal wells (24 hr later)	16	7	15	22	3	13	14	17	9	24	23	18
Time (avg | std)	25.33	4.16		36.33	11.59		22.33	2.52		31	0	
Crystals (avg | std)	12.67	4.93		12.67	9.5		13.33	4.04		21.67	3.21	

**Crystal Plate Prep**	Human	OT2
Time Average	28	31
Time Std dev	7.26	0
Crystal Average	12.89	21.67
Crystal Std dev	6.6	3.21

All data for the OT-2 vs Human HEWL protein crystallization plate preparation. The final table (lower left) contains the identical raw information to Figure 7.

Supplemental figure 6

	Person 1		Person 2		Person 3		Opentrons 2		
	mass (ug) of 1 uL	mass (ug) of 100 uL	mass (ug) of 1 uL	mass (ug) of 100 uL	mass (ug) of 1 uL	mass (ug) of 100 uL	mass (ug) of 1 uL	mass (ug) of 2 uL	mass (g) of 100 uL
Trial 1	0.9	99.1	0.8	99.6	0.9	98.7	0.8	1.6	98.3
Trial 2	0.8	98.9	0.8	99	0.9	98.6	0.9	1.7	99
Trial 3	0.8	99.3	0.8	99	1	98.6	0.8	1.8	99.1
Trial 4	0.9	99.1	0.9	99.5	0.9	98.8	1	1.7	98.8
Trial 5	0.8	98.9	0.7	99.1	0.9	99.1	0.9	1.9	98.9
average	0.84	99.06	0.8	99.24	0.92	98.76	0.88	1.74	98.82
std dev	0.05	0.17	0.07	0.29	0.04	0.21	0.08	0.11	0.31
expected val	1	100	1	100	1	100	1	2	100
MAPE	16	0.94	20	0.76	8	1.24	12	13	1.18

**1 uL water**	Human	OT2
Mean (ug)	0.85	0.88
Std dev (ug)	0.05	0.08
MAPE (%)	14.67	12

**100 uL water**	Human	OT2
Mean (ug)	99.02	98.82
Std dev (ug)	0.23	0.31
MAPE (%)	0.98	1.18

All data for the OT-2 vs Human 1 μL and 100 μL pipet comparison. Also included is the OT-2's ability to dispense 2 μL, since this is the smallest volume used in any experiment.

Supplemental Figure 7

	mass (g) of 2 uL 100% glycerol	mass (g) of 2 uL 80% glycerol	mass (g) of 2 uL 60% glycerol	mass (g) of 2 uL 40% glycerol	mass (g) of 2 uL 20% glycerol	mass (g) of 2 uL 10% glycerol
Trial 1	2.2	2.3	2	2.3	2	1.9
Trial 2	2.2	2.4	1.9	2.3	1.9	2
Trial 3	2.1	2.4	2.2	2.2	2	2
Trial 4	2.3	2.4	2.3	2	2.1	1.9
Trial 5	2.3	2.3	2.3	2.2	2	1.9
Average	2.22	2.36	2.14	2.2	2	1.94
Standard Dev	0.08	0.05	0.18	0.12	0.07	0.05
Expected	2.52	2.44	2.34	2.22	2.12	2.06
MAPE	13.51	3.39	9.35	0.91	6	6.19

	mass (g) of 100 uL 100% glycerol	mass (g) of 100 uL 80% glycerol	mass (g) of 100 uL 60% glycerol	mass (g) of 100 uL 40% glycerol	mass (g) of 100 uL 20% glycerol	mass (g) of 100 uL 10% glycerol
Trial 1	121.4	119.2	114.1	108.3	102.6	100.9
Trial 2	119.1	117.8	112.5	110.3	103.1	101.4
Trial 3	123.1	116.7	115.2	109.8	102.5	101.2
Trial 4	123.4	118.6	114.1	108.1	102.4	102.1
Trial 5	119.7	119.1	113.9	107.9	103.1	101.6
Average	121.34	118.28	113.96	108.88	102.74	101.44
Standard Dev	1.94	1.04	0.96	1.09	0.34	0.45
Expected	126	122	117	111	106	103
MAPE	3.84	3.15	2.67	1.95	3.17	1.54

All data for the OT-2 pipetting of various Glycerol dilutions (v/v), using both a 2 μL and 100 μL transfer volume.
